# Nanotoxicology: in Vitro–in Vivo Dosimetry

**DOI:** 10.1289/ehp.1104320

**Published:** 2012-01-01

**Authors:** Günter Oberdörster

**Affiliations:** University of Rochester, Department of Environmental Medicine, Rochester, New York, E-mail: gunter_oberdorster@urmc.rochester.edu

Gangwal et al. (2011) addressed an important topic for nanotoxicology about assessing the toxicity of inhaled nanomaterials by recommending relevant concentrations for *in vitro* toxicity testing. Their efforts to select *in vitro* concentrations based on reported occupational exposure levels of inhaled nanomaterials are, indeed, laudable. Their underlying conceptual approach is logical, involving a widely used and well-accepted particle dosimetry model [multiple-path particle dosimetry (MPPD)] to estimate deposited and retained mass doses in the pulmonary alveolar region of nanomaterial-exposed workers. They then expressed these doses as per-unit alveolar surface area in order to select for *in vitro* testing the same alveolar epithelial cell surface area doses. However, while this concept makes good sense when applying it to short-term (daily) deposited doses, it makes less sense and can be highly misleading when the same approach is used for doses that have accumulated in the alveolar region after long-term chronic inhalation exposures of humans. Thus, it is unfortunate that the authors made it a main point to estimate (although crudely and with some questionable assumptions) the dose of inhaled nanomaterials that is retained or accumulated on the pulmonary alveolar surface over a full working lifetime of 45 years of exposure to 1 mg/m^3^ airborne concentration. Gangwal et al. then converted this 45-year accumulated surface area dose to an equivalent *in vitro* concentration (per square centimeter) as a selection criterion for *in vitro* dosing. Under “Concentrations recommended for *in vitro* testing,” they concluded that the long-term retained human alveolar surface area dose equates to *in vitro* concentrations of 50–68 µg/mL and that

These amounts for a full working lifetime lie within the range of the highest *in vitro* assay concentrations tested in the literature for Ag [silver nanoparticles] and TiO_2_ [titanium dioxide nanoparticles] on human, rat, and mouse cell lines. (Gangwal et al. 2011)

These are extraordinarily high concentrations, and unfortunately this article may be viewed as a justification for using such high *in vitro* dosing uncritically. Gangwal et al. (2011) did not discuss anywhere in the article the reasoning behind equating lifetime accumulated doses with doses that are given all at once as a bolus in a short-term *in vitro* system. The difference in dose rate alone—not considering anything else—spans many orders of magnitude. At best, these extrapolated high *in vitro* concentrations may be labeled as the high-end limit of an *in vitro* study using a wide range of doses.

To their credit, Gangwal et al. (2011) estimated lung surface area doses achieved for a 24-hr exposure to an inhaled concentration of 1 mg/m^3^ and—as one would expect—extrapolated this to much lower concentrations of 0.17–0.57 µg/mL for equivalent *in vitro* dosing with TiO_2_ and Ag nanoparticles. If they had used a more realistic higher value for the human alveolar surface area—as they did for the full working lifetime exposure—the extrapolated equivalent short-term *in vitro* concentrations would have been even lower by about one order of magnitude. Unfortunately, the authors did not emphasize the tremendous differences between actual high doses used in most published *in vitro* studies of nanoparticles and the more realistic much lower *in vitro* doses. For *in vitro* testing, use of a wide range of doses, starting at—or even better—below the 24-hr inhalation equivalent and increasing to a maximum of the lifetime exposure equivalent, could be a practical approach.

With respect to carbon nanotubes (CNTs), Gangwal et al. (2011) reported results only for the full working lifetime exposure scenario and the resulting extrapolated equivalent *in vitro* concentrations. According to the authors, these extrapolated high equivalent *in vitro* concentrations are at the low end of concentrations that have been reported for CNTs in the *in vitro* literature. Implications for selection of realistic *in vitro* exposures to CNTs were not discussed, nor was the more relevant 24-hr exposure scenario for CNTs modeled to derive an equivalent short-term *in vitro* dose. This would have provided a suggested range of *in vitro* dosing for CNTs as pointed out above for Ag and TiO_2_ nanoparticles, provided the dosimetry model (MPPD) is applicable for CNTs. Unfortunately, the validity of the MPPD model for fiber-shaped structures of nanosized dimensions was neither explained in sufficient detail by Gangwal et al., nor has it been confirmed and published, specifically for nanofibers and nanotubes. Moreover, a thorough literature search reveals that CNT aerosols at workplaces are not present as individual straight nanofibers, but occur mostly as small and large tangles of different shapes of hollow tubes with unknown effective density (density is not that of solid carbon) (Han et al. 2008; Methner et al. 2010; Tsai et al. 2009). There is currently no deposition model that could be applied for such nanostructures without additional research to obtain necessary input data.

A careful selection of *in vitro* doses for nanoparticle toxicity testing is very important. Thus, authors, reviewers, and journal editors should be critical when submitting, reviewing, and accepting papers for publication.
